# Brain Control of Olfaction via Top-down Regulation on Adult Neurogenesis

**DOI:** 10.3389/fnins.2012.00117

**Published:** 2012-08-06

**Authors:** Livio Oboti, Jean-Claude Platel

**Affiliations:** ^1^Department of Physiology, University of SaarlandHomburg, Germany; ^2^Grenoble Institute des Neurosciences U836, Université Joseph Fourier GrenobleGrenoble, France

According to Dr. Pangloss (Voltaire, 1759), noses were made to wear glasses, legs were meant to wear pants, and olfactory bulb newborn granule cells (GCs) to improve olfaction. While the first two hypotheses have been rejected almost unanimously, the latter may not be far from the truth, yet the reason has not been defined. Initially, this assumption has emerged by revealing the physiological properties of newborn GCs: they are highly excitable and preferentially involved in events of synaptic plasticity (Nissant et al., 2009), they selectively respond to experienced stimuli (Magavi et al., 2005), their ablation impairs olfactory learning and memory (see for example Mouret et al., 2008; Moreno et al., 2009; Oboti et al., 2011) while their specific stimulation facilitates these processes (Alonso et al., 2012). Inspired by the definition of the *receptor neuron-to-mitral* *cell* labeled-line as the essential functional unit of the OB circuitry, most functional studies have analyzed the problem under a paradigm dictated by this centripetal logic: a manipulation in the periphery (environment, or local OB circuitry) is used to test the central brain function. However, GCs connectivity is not limited to local neuronal networks (interneurons and mitral cells). These cells also receive centrifugal inputs onto their basal and proximal dendritic domains and show early synaptic responses to glutamate before the establishment of synaptic connections with mitral cells at the more distal portions of their dendritic arbors (Panzanelli et al., 2009). In addition, olfactory deprivation differentially affects spine density in the distal and proximal dendritic domains, where respectively centripetal and centrifugal synaptic connections take place (Kelsch et al., 2009). Hence, the role of central brain activity is exceedingly likely to affect the fate of developing GCs, but the mechanisms of this effect have yet to be elucidated. Surprisingly, only a few reports have aimed to do so (Bauer et al., 2003; Cooper-Kuhn et al., 2004; Veyrac et al., 2005, 2009) and, although focusing on the local OB reveals why younger neurons may be preferable to older ones, the mechanisms by which the brain makes this choice can not be fully clarified without considering its feedback modulatory activity.

Moreno et al. (2012) have recently shed new light on this issue and analyzed the interaction between centrifugal innervation and plasticity in the olfactory bulb in a sensory context meaningful for both. The authors investigated the involvement of the noradrenergic signaling in the olfactory perceptual learning, a form of learning in which previous experience like olfactory habituation allows discrimination between perceptually similar odorants.

They showed that blocking the noradrenergic system during olfactory enrichment abolishes its positive effects on olfactory discriminatory ability while the stimulation of the noradrenergic system alone results in better performances in the discriminatory task. To complete the picture of the involvement of the noradrenergic system, they showed that olfactory enrichment induced a higher activity in the locus coeruleus, the source of noradrenergic fibers in the brain. Previous reports revealed that perceptual learning requires the incorporation and activation of newborn GCs (Moreno et al., 2009). Consistently, treatment with an agonist of the noradrenergic signaling increases newborn GCs survival, mimicking the effects of olfactory enrichment. These results, in conjunction with other studies on cholinergic or glutamatergic centrifugal afferents (Cooper-Kuhn et al., 2004; Oboti et al., 2011), suggest that the selection of newborn elements in this peripheral sensory circuitry depends on the concurrence of olfactory inputs and the consequent backward stimulation from downstream brain nuclei. This eventually results in neuronal activation and survival in those OB regions precisely and specifically matched with the activity patterns evoked by this concerted sensory stimulation. Moreno et al. give credence to this hypothesis by showing that familiarized odorants induce higher activity in newborn cells (% BrdU+/Zif268+) under enriched conditions, an effect that is abolished when the noradrenergic blocker is given during the olfactory enrichment protocol. This suggests that centrifugal brain activity alone (in this case noradrenergic) represents an important factor in determining the extent of OB neuronal excitability, an effect particularly evident in the newborn GCs. It would be interesting in the future to determine if the spatial mapping of BrdU/Zif268-positive cells match the activity pattern of familiarized odors or if it is more widespread. A detailed screening of NA-receptor expression in these cells along different maturational steps would certainly sustain this interpretation and give a more precise view of their functional integration dynamics. However, limited by the experimental paradigm adopted in this work, OB neurogenesis and noradrenergic signaling seem to be tightly connected. Accordingly, ablation of younger neurons through short-term *AraC* treatment impedes olfactory enrichment to improve mice discriminatory abilities. In contrast to what happens when new neurons are still present, this defect is not rescued by the stimulation of the noradrenergic system. Eventually lesioning of the noradrenergic inputs on the OB, possibly the LC, would further confirm this observation. These results strongly suggest that olfactory perceptual learning in mice is improved specifically by the noradrenergic control of newborn cell functional integration. This direct link is further supported by the observation of synaptic contacts between young GCs and noradrenergic fibers and direct GCs response to noradrenaline stimulation.

These interesting results, combined with other studies, support the idea that the selection of functional elements in the peripheral sensory circuitry depends on the concurrence of external inputs and the consequent backward stimulation from downstream brain nuclei (Gilbert and Sigman, 2007; Fletcher and Chen, 2010; Robinson et al., 2011). This opens the way to new connectome studies focused on the role of other regions sending afferent projections to the OB, such as the anterior olfactory nucleus, the limbic system, the piriform cortex, among others. The main question should now shift from *why* individual cells are important, to *how* the whole brain uses them. Indeed, either stimulus novelty, arousal, or the rewarding value of olfactory cues, seem to define the functional context in which olfactory bulb neurogenesis is modulated (Figure 1). In the end it is true, a nose shape has nothing to do with the reason why we may need glasses, but its connections to the brain probably will tell us why its neuronal plasticity is so important.

**Figure 1:**
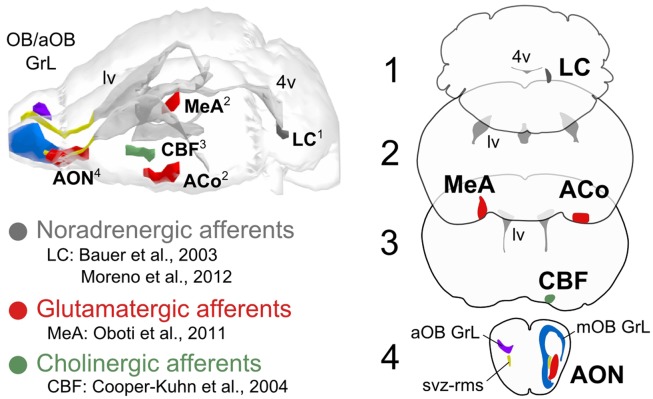
**Principal brain centrifugal afferents to the olfactory bulb**. The modulatory role of noradrenergic, glutamatergic, and cholinergic brain afferents on neuronal survival in the olfactory bulb (main and accessory, mOB/aOB) has been reported by the studies indicated by the references. The projection sites of noradrenergic (locus coeruleus, LC), glutamatergic (medial, cortical amygdala, and anterior olfactory nucleus; MeA, ACo, AON), and cholinergic afferents (cholinergic basal forebrain, CBF) to the olfactory bulb are represented unilaterally in the brain model and in the coronal planes on the right, ipsilaterally to the targeted bulbar region (mOB/aOB). Other abbreviations used: svz-rms, sub-ventricular zone-rostral migratory stream; GrL, granular layer; lv, lateral ventricle; 4v, fourth ventricle.

## References

[B1] Alonso M. Lepousez G. Wagner S. Bardy C. Gabellec M. M. Torquet N. Lledo P. M. (2012). Activation of adult-born neurons facilitates learning and memory. Nat. Neurosci. 15, 897–904 10.1038/nn.3108 22581183

[B2] Bauer S. Moyse E. Jourdan F. Colpaert F. Martel J. C. Marien M. (2003). Effects of the alpha 2-adrenoreceptor antagonist dexefaroxan on neurogenesis in the olfactory bulb of the adult rat in vivo: selective protection against neuronal death. Neuroscience 117, 281–291 10.1016/S0306-4522(02)00757-1 12614670

[B3] Cooper-Kuhn C. M. Winkler J. Kuhn H. G. (2004). Decreased neurogenesis after cholinergic forebrain lesion in the adult rat. J. Neurosci. Res. 77, 155–165 10.1002/jnr.20116 15211583

[B4] Fletcher M. L. Chen W. R. (2010). Neural correlates of olfactory learning: critical role of centrifugal neuromodulation. Learn. Mem. 17, 561–570 10.1101/lm.941510 20980444 PMC2981412

[B5] Gilbert C. D. Sigman M. (2007). Brain states: top-down influences in sensory processing. Neuron 54, 677–696 10.1016/j.neuron.2007.05.019 17553419

[B6] Kelsch W. Lin C. W. Mosley C. P. Lois C. (2009). A critical period for activity-dependent synaptic development during olfactory bulb adult neurogenesis. J. Neurosci. 29, 11852–11858 10.1523/JNEUROSCI.2406-09.2009 19776271 PMC2773669

[B7] Magavi S. S. Mitchell B. D. Szentirmai O. Carter B. S. Macklis J. D. (2005). Adult-born and preexisting olfactory granule neurons undergo distinct experience-dependent modifications of their olfactory responses in vivo. J. Neurosci. 25, 10729–10739 10.1523/JNEUROSCI.2250-05.2005 16291946 PMC6725839

[B8] Moreno M. M. Bath K. Kuczewski N. Sacquet J. Didier A. Mandairon N. (2012). Action of the noradrenergic system on adult-born cells is required for olfactory learning in mice. J. Neurosci. 32, 3748–3758 10.1523/JNEUROSCI.6335-11.2012 22423095 PMC6703441

[B9] Moreno M. M. Linster C. Escanilla O. Sacquet J. Didier A. Mandairon N. (2009). Olfactory perceptual learning requires adult neurogenesis. Proc. Natl. Acad. Sci. U.S.A. 106, 17980–17985 10.1073/pnas.0900701106 19815505 PMC2764902

[B10] Mouret A. Gheusi G. Gabellec M. M. de Chaumont F. Olivo-Marin J. C. Lledo P. M. (2008). Learning and survival of newly generated neurons: when time matters. J. Neurosci. 28, 11511–11516 10.1523/JNEUROSCI.2954-08.2008 18987187 PMC6671302

[B11] Nissant A. Bardy C. Katagiri H. Murray K. Lledo P. M. (2009). Adult neurogenesis promotes synaptic plasticity in the olfactory bulb. Nat. Neurosci. 12, 728–730 10.1038/nn.2298 19412168

[B12] Oboti L. Schellino R. Giachino C. Chamero P. Pyrski M. Leinders-Zufall T. Zufall F. Fasolo A. Peretto P. (2011). Newborn interneurons in the accessory olfactory bulb promote mate recognition in female mice. Front. Neurosci. 5:113 10.3389/fnins.2011.00113 21994486 PMC3182443

[B13] Panzanelli P. Bardy C. Nissant A. Pallotto M. Sassoè-Pognetto M. Lledo P. M. Fritschy J. M. (2009). Early synapse formation in developing interneurons of the adult olfactory bulb. J. Neurosci. 29, 15039–15052 10.1523/JNEUROSCI.3034-09.2009 19955355 PMC6665970

[B14] Robinson L. Platt B. Riedel G. (2011). Involvement of the cholinergic system in conditioning and perceptual memory. Behav. Brain Res. 221, 443–465 10.1016/j.bbr.2011.01.055 21315109

[B15] Veyrac A. Didier A. Colpaert F. Jourdan F. Marien M. (2005). Activation of noradrenergic transmission by alpha 2-adrenoceptor antagonists counteracts deafferentation-induced neuronal death and cell proliferation in the adult mouse olfactory bulb. Exp. Neurol. 194, 444–456 10.1016/j.expneurol.2005.03.004 16022870

[B16] Veyrac A. Sacquet J. Nguyen V. Marien M. Jourdan F. Didier A. (2009). Novelty determines the effects of olfactory enrichment on memory and neurogenesis through noradrenergic mechanisms. Neuropsychopharmacology 34, 786–795 10.1038/npp.2008.191 18946468

[B17] Voltaire J. (1759).Candide, ou l’Optimisme. eds Cramer Rey Marc-Michel Nourse Jean Lambert others.

